# Bilateral Anterior Cruciate Ligament Reconstruction Using Gracilis and Semitendinosus Graft by Single-Staged Approach

**DOI:** 10.7759/cureus.46038

**Published:** 2023-09-26

**Authors:** Adarsh Jayasoorya, Ankur Salwan, Amit Saoji, Kevin Kawde

**Affiliations:** 1 Department of Orthopaedics, Jawaharlal Nehru Medical College, Datta Meghe Institute of Higher Education and Research, Wardha, IND; 2 Department of Orthopaedic Surgery, Jawaharlal Nehru Medical College, Datta Meghe Institute of Higher Education and Research, Wardha, IND

**Keywords:** arthroscopy, knee joint, ligament reconstruction, anterior cruciate ligament, acl

## Abstract

A bilateral anterior cruciate ligament (ACL) tear is one of the rare injuries that is seen in orthopaedics practice. Although few single-staged bilateral ACL ruptures have also been documented, most bilateral ACL ruptures happen on two different occasions. Although there isn't a clear consensus, there have been accounts of both single-staged and two-staged reconstruction of bilateral ACL ruptures in the literature. This case report provides surgeons with options to consider while treating this unusual injury. A 35-year-old woman with bilateral anterior cruciate ligament injuries presented with an MRI of her left knee suggestive of a complete ACL tear with a Medial meniscus tear in the left knee, and an MRI of her right knee showed a complete ACL tear. The patient underwent arthroscopic ACL reconstruction in a single stage for both knees. Six months after her surgery, she had met all the rehabilitation goals and was cleared to resume her daily activities. The patient preoperatively had a visual analogue scale (VAS) score of 8, and postoperative assessment, her VAS score reduced to 2. ACL reconstruction in one stage and two stages were the surgical treatment modalities described in the literature. Concurrent rehabilitation of both ACL repairs is more economical, reduces hospital stay, and helps in early recovery, but it may result in severe quadriceps deconditioning. Double-staged surgeries are less demanding, with a shorter duration of surgery that can be performed by a less experienced surgeon. As single-staged bilateral ACL reconstruction is a less expensive option that reduces hospital stays and aids in early recovery for this rare patient population, it may be a great therapy option compared to two-staged bilateral ACL reconstruction.

## Introduction

Primary and secondary constraints are provided by the anterior cruciate ligament (ACL) for the internal tibial rotation and anterior tibial translation, respectively [1]. An ACL tear can result in knee instability, which can harm the meniscus, cause articular cartilage to deteriorate, and put more strain on surrounding soft tissues. The ACL is most frequently injured during sports activities, but it can also be torn during work-related accidents and other non-athletic pursuits. The reported annual incidence of an ACL injury in the general population ranges from 0.01% to 0.08%. However, athletes who participate in multidirectional sports experience a substantially higher incidence (1.5% to 1.7%) [2,3]. An incidence of 2-4% indicates that bilateral ACL rupture is a relatively uncommon occurrence [4,5]. Although rare examples of single-staged bilateral ACL ruptures have been documented, most bilateral ACL ruptures happen on two separate occasions. Non-operative management of ACL rupture may improve the injured knee's perception of "stability," but not at the expense of objective result evaluation or rate of return to sports [6,7]. It has been demonstrated that arthroscopic reconstruction of the injured ACL can successfully restore knee stability, prevent additional knee deterioration, and improve the return to complete activities and high-level sporting activities as they were before the injury. Without a clear consensus, there have been accounts of both single-staged and two-staged reconstruction of bilateral ACL ruptures in the literature. The main concern is that serious quadriceps deconditioning could result from simultaneous rehabilitation of double ACL surgeries and prolonged duration. In this case report, we discuss the case of a 35-year-old patient who underwent double-stage bilateral knee arthroscopic ACL surgeries for bilateral ACL tears. The patient was explained and gave her permission for the publication of her case-related information.

## Case presentation

A 35-year-old female came to the orthopedics outpatient department at Acharya Vinoba Bhave Rural Hospital (AVBRH) Sawangi, Wardha, complaining of pain and instability in her right knee for two years (July 2019). Pain and instability over left knee for one month (June 2021). The patient alleged a history of a twisting injury to her right knee two years back and a history of slip and fall one month back while working at home, sustaining an injury to both knees. Immediately after the trauma, she developed pain and swelling over both knees. The pain was sudden in onset, gradually progressive, and dull aching type, which got aggravated by walking, and movements and relieved by taking rest and medication. Earlier, he developed instability over the right knee and was walking without support. Still, the patient was unable to do brisk walking. Presently, she has instability in the left knee while walking on uneven surfaces. The patient sought initial care at a private hospital, where she experienced pain relief. The patient then came to AVBRH for further management.

**Figure 1:**
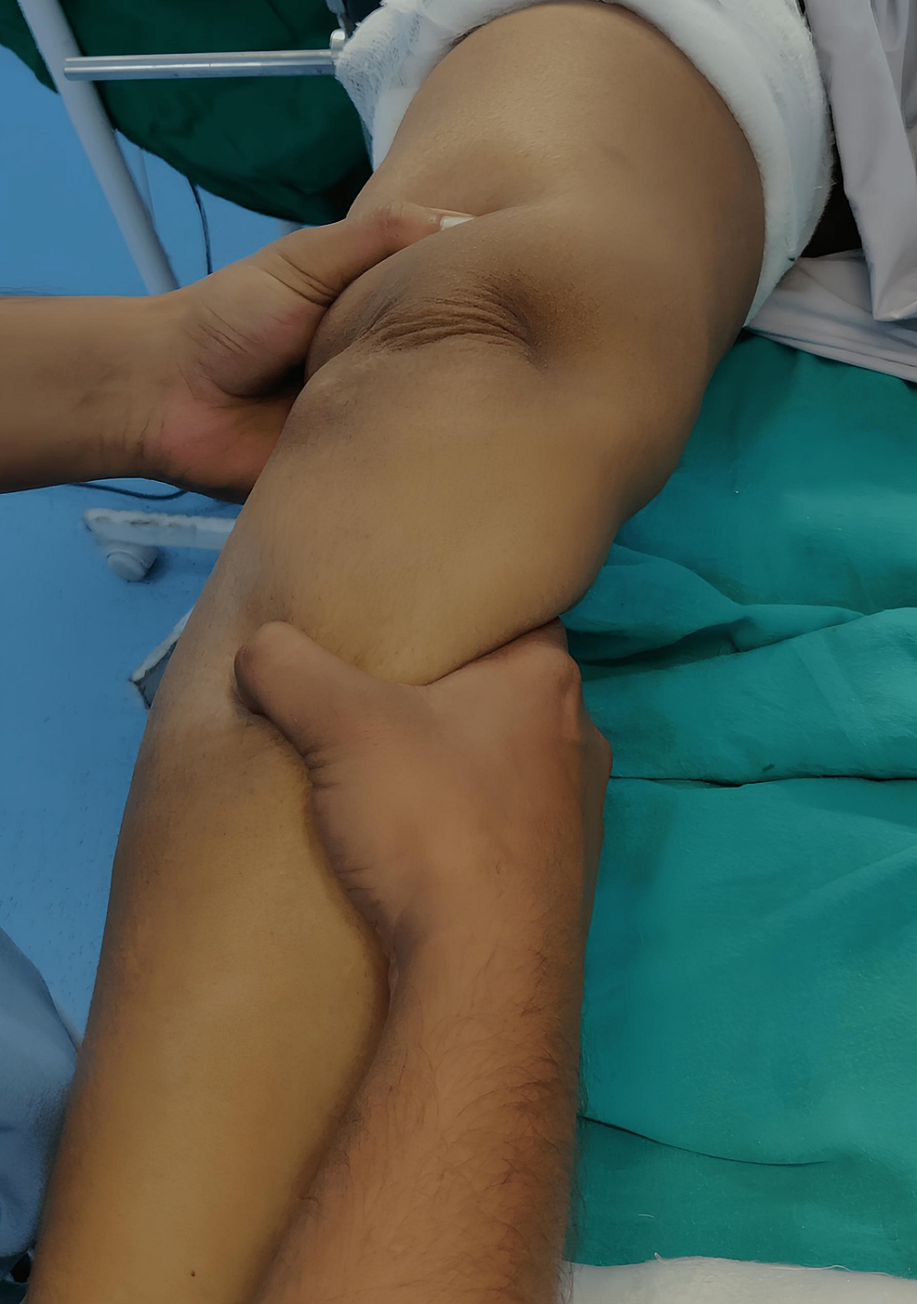
Clinical image showing a positive anterior drawer test on right knee

On Examination of the right knee, it was evident that there was no obvious abnormality. On palpation of the right knee, the knee range of motion (ROM) was full, the anterior drawer test was positive, and Lachman's test was positive (Figure 1).

**Figure 2:**
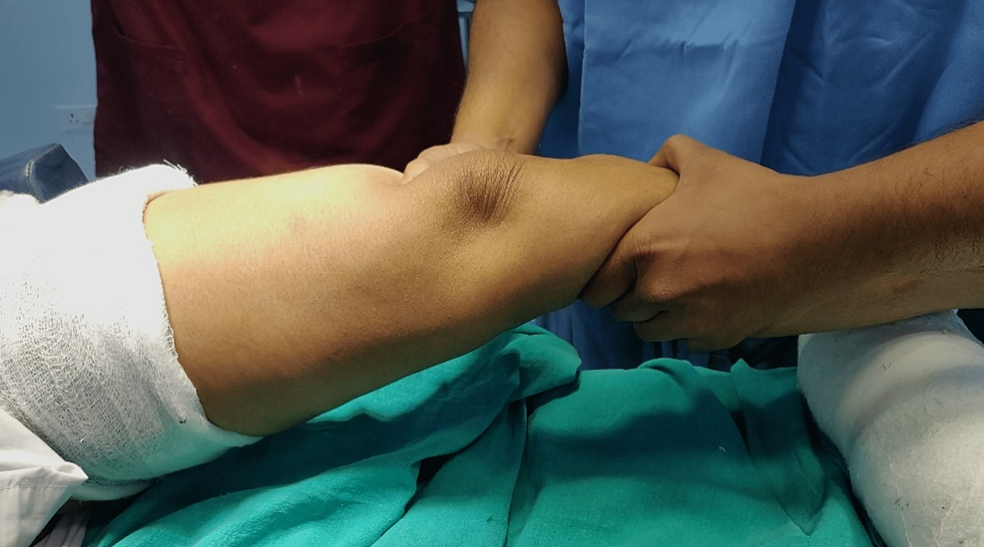
Clinical image showing positive anterior drawer test on left knee

On Inspection of the left knee: Inspection relieved no obvious abnormalities. On palpation of the left knee, tenderness was found to be present over the medial joint line, the knee ROM was full, the anterior drawer test was positive, the Lachmans test was positive (Figure 2), Mc Murrays test and Thessaly tests were positive for medial meniscus. On examination, the patient was also found to have increased generalized laxity and had a Beighton score of 7/9 points.

**Figure 3:**
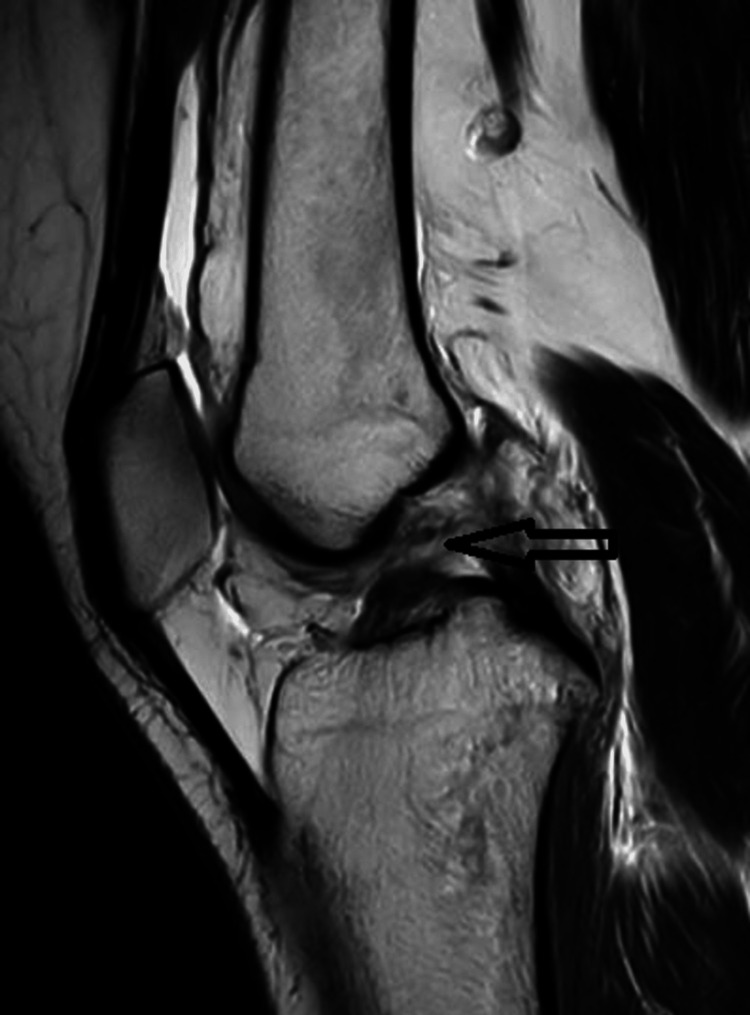
Left knee MRI image The image shows a complete ACL tear with a medial meniscus tear.

Preoperative radiographs, including X-ray bilateral knee AP (anteroposterior) and lateral view, showed no obvious bony abnormality; MRI of the left knee showed complete ACL tear left knee with medial meniscus tear left knee with multiple small chondral bodies in the anterior lateral gutter, the largest measuring 7 mm (Figure 3). 

**Figure 4:**
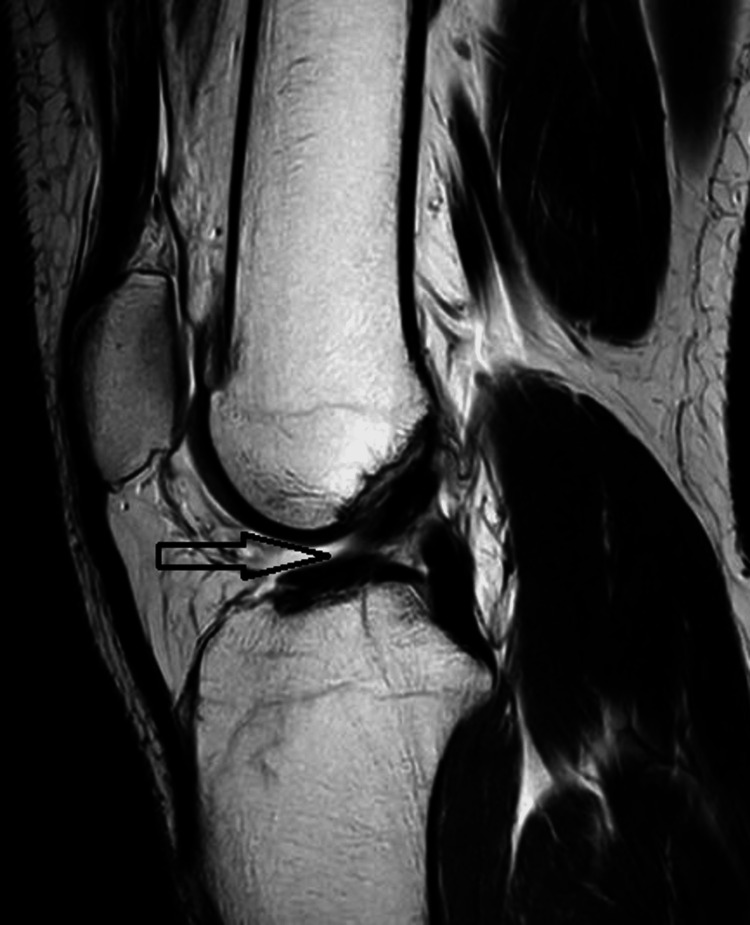
Right knee MRI image The image shows a complete ACL tear in the right knee.

MRI of the right knee shows a complete ACL tear (Figure 4). In view of her young age and the activities she wanted to undertake, it was decided that she would probably need both ACLs reconstructed in addition to any meniscal surgery. Treatment options discussed included conservative and operative management.

**Figure 5:**
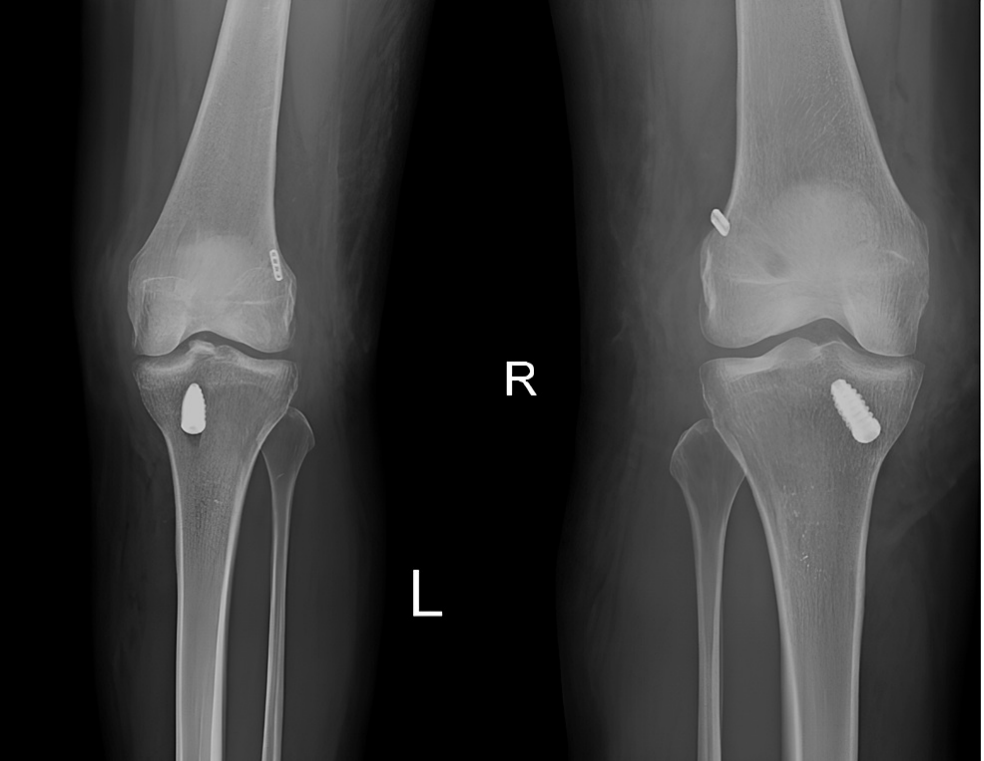
Post-operative X-ray bilateral knee AP view AP: Anterior–posterior

**Figure 6:**
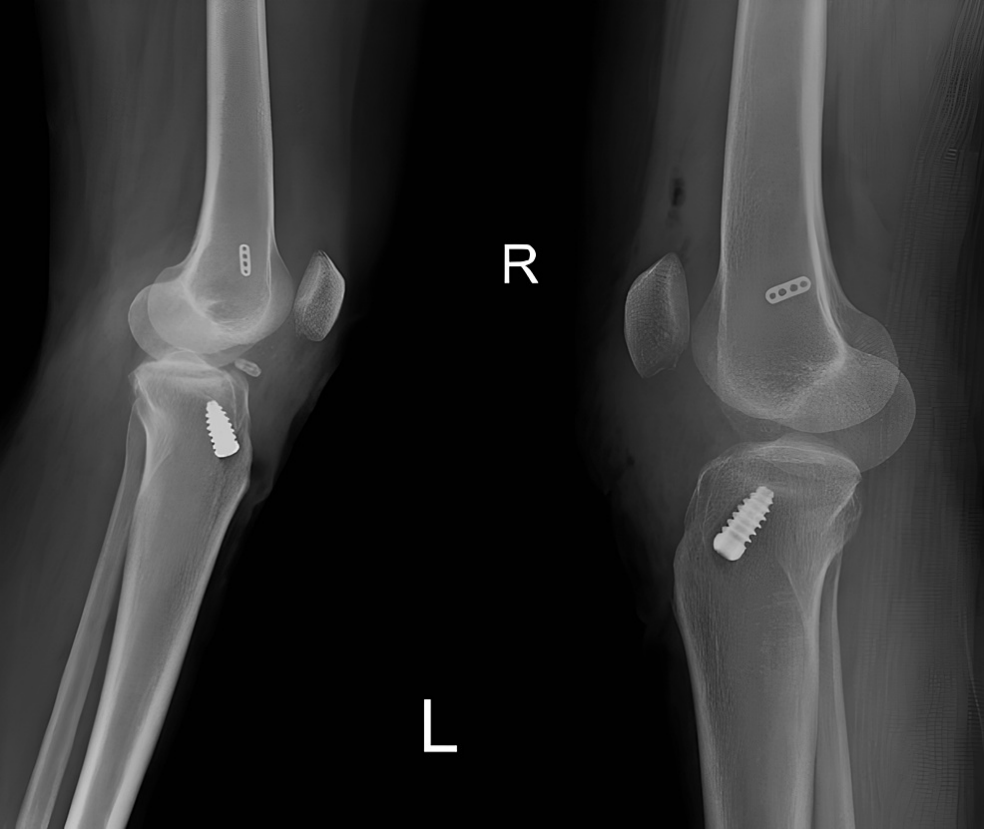
Post-operative X-ray bilateral knee lateral view

The patient was suggested for a single-staged bilateral knee procedure. Under spinal plus epidural anesthesia, the bilateral lower limb was cleaned, painted, and draped. A tourniquet was inflated to 300 mm of Hg on the left lower limb. A 3 cm incision was made approximately 2 cm distal and medial to the tibial tuberosity. Hemostasis was achieved. Semitendinosus tendon and gracilis tendon were identified and separated, and a graft was harvested using a tendon stripper. Anterolateral and anteromedial portals were made and scope was inserted. The medial and lateral gutter was found to be expected. Synovial hypertrophy was noted and was shaved off. A medial meniscus bucket handle tear was noted, and the posterior cruciate ligament (PCL) was intact. Impinging part of the meniscus was shaved off, and medial meniscectomy was done. ACL tear and insufficiency were visualized, and remnants of ACL were shaved off. Tibia and femoral tunnels were made appropriately at the footprint, and the tunnel was prepared with a 7 mm drill. An endobutton loop of 15 mm was used. Graft measuring 9 cm inserted and fixed with interference screw 8 x 20 mm. A thorough wash was given with normal saline. The closure was done in layers with vicryl 0 and ethilon 2-0. Similarly, ACL was reconstructed on the right lower limb, after which dressing was applied with Robert Jones (RJ) bandage and crepe compression was given. A long knee brace was applied. The procedure was uneventful, and the patient was shifted to post-op recovery. Physiotherapy in the form of a closed chain range of movement of bilateral knees from 0-30 degrees was started post-operative day 1. On post-operative day 2, surgical site dressing was done, and the suture site was found to be healthy and healing. A post-operative X-ray was done on day 2 and was found to be satisfactory (Figures 5-6).

**Figure 7:**
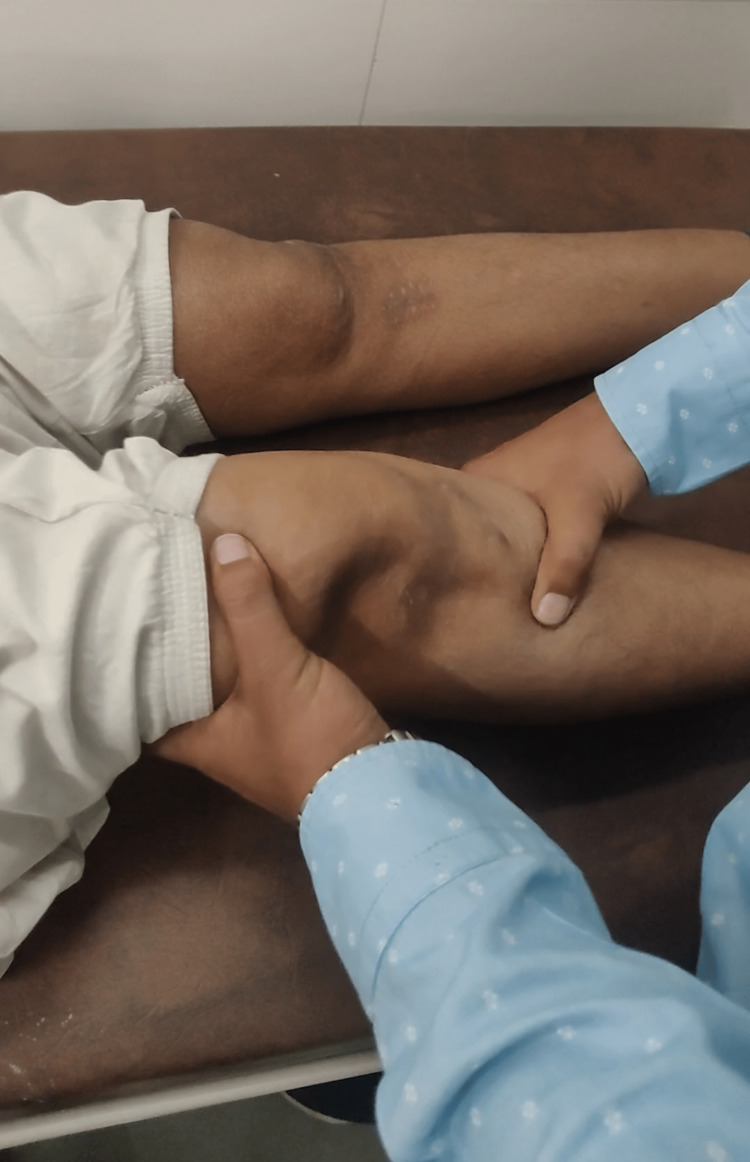
Postoperative clinical image The image shows a healthy suture site and negative anterior drawer test bilaterally.

Anterior drawers test was found to be negative on the bilateral lower limb during the first review (Figure 7).

Full weight-bearing mobilization was started from day 7 post-operation. From the second week onwards, a 30-100 degree range of movement was started, and the full range of motion was achieved after six weeks.

## Discussion

The increased participation of people of all ages and genders in sports may be why the anterior cruciate ligament is one of the ligaments that most commonly rupture. People between the ages of 15 and 25 who play contact sports are at the highest risk of ACL rupture. With a reported prevalence of 2%-4% [4,5], bilateral ACL injury is quite unusual. The majority of these injuries happen at various periods. Bilateral ACL tears that occur in one stage are extremely uncommon, and most instances recorded in the literature are unique, isolated occurrences. Contralateral ACL injury's risk factors, etiology, and mechanisms are not as well-established and definitive in the literature as they are for unilateral ACL damage. The preferred course of treatment for ACL injuries is ACL reconstruction, particularly in young, athletic individuals who are most susceptible to this condition. Early resumption of pre-injury activity levels is the aim of reconstruction. The best reconstructive methods and rehabilitation programs for unilateral ACL injuries are thoroughly described in the literature. Although patients with bilateral ACL rupture can be treated with either a single-staged or two-staged ACL reconstruction, there is still no agreement on how to treat these patients. ACL restoration procedures involving one or two stages have unique benefits and drawbacks. Single-stage surgery is both time- and cost-efficient because it dramatically lowers overall direct surgical expenses and may also result in fewer sick days, quicker recovery times, and less opioid use [8,9], although it may cause reduced activity post-operatively to the point that severe quadriceps weakness develops despite vigorous rehabilitation programs [10]. Additionally, there is considerable worry regarding an elevated likelihood of re-injury following single staged bilateral postoperative functional knee impairment. Whereas two staged bilateral ACL restoration offers the advantages of being a less difficult surgery, taking less time to complete even though it may cause higher hospital costs, more extended hospital stay, and increased time required for recovery. So, deciding which surgical management is still up for dispute will require more studies for a better understanding.

## Conclusions

ACL injuries on both sides are relatively uncommon and more frequently result from two independent injuries than a single accident in patients with symptomatic bilateral ACL deficient knees. Single-staged bilateral ACL reconstruction significantly reduces direct surgical costs and saves time and money. Additionally, it also helps for fewer sick days, quicker recoveries, and reduced opioid use and thus is a suitable surgical option for these rare patients even though there is a chance of quadriceps deconditioning. This case demonstrates that single-staged bilateral arthroscopic ACL reconstruction is a potentially effective surgical approach for a patient with bilateral proximal ACL tears.
